# Einsteins' role in clinical research

**DOI:** 10.1590/S1679-45082014ED3063

**Published:** 2014

**Authors:** Nelson Wolosker, Luiz Vicente Rizzo, Cláudio Schvartsman

**Affiliations:** 1 Hospital Israelita Albert Einstein, São Paulo, SP, Brazil

Scientific research is a systematic process aimed to build knowledge through a set of structured evaluations, thus generating the development of both the individual that performs research and the society in which it is developed.

The objective of research is to produce knowledge, possibly applicable inside and outside researchers' environments, and perhaps generate practical actions. Research methodology should be reproducible and its results may be taken into real world scenarios, generating outcomes for the entire community.

A good example of this process was the beginning of the past decade, when patients with ischemia that presented a short stenosis of the superficial femoral artery were treated with saphenous bypass surgery attaining very good results, but at the expense of high clinical complication rates. Several studies using angioplasty associated with stent placements demonstrated that the same lesions had appropriate long-term results, and lower complication rates.^(1,2)^ This new piece of information and cost-effectiveness-based concepts changed management, and patients were no longer submitted to major surgery, and began to be treated by the endovascular method.

A well conducted study is considered the foundation for the development of a good health policy in any part of the world. Instead of only relying on what in theory could be effective or work, or use empirical or political methods, investigators perform studies that provide institutions and society with material data that will make their decisions easier, and generate effective institutional and even global health programs.

Until a few years ago, patients with hyperhidrosis were treated as fast as possible with thoracic sympathectomy, but also had a constant side effect - compensatory hyperhidrosis, which frequently generated more suffering to patients than the initial condition.^(3,4)^ Today, patients with such a frequent condition regardless of age, are initially treated with a low cost drug, oxybutynin chlorhydrate, and those who improve remain on the medication with a record of major improvement in Quality of Life; only those who do not improve are submitted to surgery.^(5,6)^ This has led to a great decrease in the number of individuals operated on, with cost reduction for payment sources and decrease in the number of irreversible and unnecessary complications.

Today, the *Hospital Israelita Albert Einstein* (HIAE) is a leader in health care, thanks to its professionals and to knowledge, especially coming from good quality Brazilian universities.

The HIAE Study Center was founded over 30 years ago, designed to join physicians of the institution for discussing cases and clinical updating. At the time no one thought of performing clinical research at the institution. The leaders believed then that research should be performed at the university, and not at a care-oriented hospital. They did not imagine that patients would consent to take part in scientific study in a private environment.

Throughout the years, HIAE grew and evolved. Like most major world institutions, research began to be a natural development, increasingly more frequent, and increasingly encouraged. We know that in the United States, major medical institutions, such as Cleveland Clinic, Mayo Clinic and others, have balanced care and research activities, in that the combination of both at a high level is responsible for their excellent worldwide reputation. Bearing this in mind, we created the Teaching and Research Institute during the year 2000 to foster the progress of scientific research at HIAE, generating exponential growth in our research activities.

The final event of a scientific study is its disclosure by publishing the study in a journal or possible development of patents. Both actions make institutions acknowledged and respected directly in the global community. With our new stand, we have invested a lot of energy in research, and in 2013, we published 198 original articles in indexed journals with an impact factor greater than 1, and received 593 citations. This happened after receiving the SciVal Award from Elsevier publishers in 2012, in collaboration with the Coordination for the Improvement of Higher Level Education Personnel (CAPES), for being the research institute in Brazil with the best average of citations per article from Brazil in the previous three-year period.

Indirectly, but not less important, research has resulted in the creation of differentiated work positions. In our case, part of the investigators is already working in different areas of care of our institution and undoubtedly would like to take part more intensely in research activities. Young professionals can evolve, can be engaged more and in a better way, in addition to increasing their credibility in the market.

Research requires structure, major planning, training and team work. In this way, it is a process based on interaction among individuals and on ongoing assessment of issues related to objectives, methods and data, which lead to new ideas, revisions and improvement of processes.

A good connection between researchers and heads of institutions makes results become of great practical importance, improving activities in all strategic niches.

A simplistic alternative to the movement in we which we find ourselves could be the maintenance of the s*tatus quo*, in which we would continue to take advantage of professionals from other institutions and of the knowledge produced by other services. This would put us in a safe and stable status in the short and medium term; but, in the long run, we would incur in the risk of involution.

Therefore, we have no doubt of our objectives: we currently and will continue to make major investments in good quality scientific research, to become an even greater entity and generate benefits to the entire Brazilian society. In this sense, we will begin, this year, our Master and PhD program in Health Sciences, approved by CAPES at level 4. Oriented toward complete development of investigators, we have innovated the subjects grid, as to setting goals and responsibilities, and in the integration among research groups and between undergraduate and graduate programs. It will be one more challenge toward the consolidation of scientific research as an integrated and essential part of our activities.
